# A Diagnostic Approach in Large B-Cell Lymphomas According to the Fifth World Health Organization and International Consensus Classifications and a Practical Algorithm in Routine Practice

**DOI:** 10.3390/ijms252313213

**Published:** 2024-12-09

**Authors:** Magda Zanelli, Francesca Sanguedolce, Maurizio Zizzo, Stefano Ricci, Alessandra Bisagni, Andrea Palicelli, Valentina Fragliasso, Benedetta Donati, Giuseppe Broggi, Ioannis Boutas, Nektarios Koufopoulos, Moira Foroni, Francesca Coppa, Andrea Morini, Paola Parente, Valeria Zuccalà, Rosario Caltabiano, Massimiliano Fabozzi, Luca Cimino, Antonino Neri, Stefano Ascani

**Affiliations:** 1 Pathology Unit, Azienda USL-IRCCS di Reggio Emilia, 42123 Reggio Emilia, Italy; stefano.ricci@ausl.re.it (S.R.); alessandra.bisagni@ausl.re.it (A.B.); andrea.palicelli@ausl.re.it (A.P.); moira.foroni@ausl.re.it (M.F.); 2 Pathology Unit, Policlinico Riuniti, University of Foggia, 71122 Foggia, Italy; francesca.sanguedolce@unifg.it; 3 Surgical Oncology Unit, Azienda USL-IRCCS di Reggio Emilia, 42123 Reggio Emilia, Italy; maurizio.zizzo@ausl.re.it (M.Z.); andrea.morini@ausl.re.it (A.M.); massimiliano.fabozzi@ausl.re.it (M.F.); 4 Laboratory of Translational Research, Azienda USL-IRCCS di Reggio Emilia, 42123 Reggio Emila, Italy; valentina.fragliasso@ausl.re.it (V.F.); benedetta.donati@ausl.re.it (B.D.); 5 Department of Medical and Surgical Sciences and Advanced Technologies “G.F. Ingrassia” Anatomic Pathology, University of Catania, 95123 Catania, Italy; giuseppe.broggi@phd.unict.it (G.B.); rosario.caltabiano@unict.it (R.C.); 6 Second Department of Pathology, Medical School, National and Kapodistrian University of Athens, Attikon University Hospital, 15772 Athens, Greece; iboutas@med.uoa.gr (I.B.); nkoufo@med.uoa.gr (N.K.); 7 Pathology Unit, Azienda Ospedaliera Santa Maria di Terni, University of Perugia, 05100 Terni, Italy; f.coppa@aospterni.it (F.C.); s.ascani@aospterni.it (S.A.); 8 Pathology Unit, Fondazione IRCCS Casa Sollievo della Sofferenza, San Giovanni Rotondo, 71013 Foggia, Italy; p.parente@operapadrepio.it; 9 Pathology Unit, Dipertimento di Patologia Umana Dell’Adulto e Dell’Età Evolutiva, Ospedale Gaetano Barresi, Università degli Studi di Messina, 98121 Messina, Italy; valeria.zuccala@unime.it; 10 Ocular Immunology Unit, Azienda USL-IRCCS di Reggio Emilia, 42123 Reggio Emilia, Italy; luca.cimino@ausl.re.it; 11 Department of Surgery, Medicine, Dentistry and Morphological Sciences, University of Modena and Reggio Emilia, 41125 Modena, Italy; 12 Scientific Directorate, Azienda USL-IRCCS di Reggio Emilia, 42123 Reggio Emilia, Italy; antonino.neri@ausl.re.it

**Keywords:** large B-cell lymphoma, high-grade B-cell lymphoma, Burkitt lymphoma, diffuse large-B-cell lymphoma, not otherwise specified, *MYC* rearrangement, *BCL6* rearrangement, *BCL2* rearrangement, *IRF4* rearrangement, *11q* aberrations

## Abstract

In this article, we provide a review of large B-cell lymphomas (LBCLs), comparing the recently published fifth edition of the WHO classification and the International Consensus Classification (ICC) on hematolymphoid tumors. We focus on updates in the classification of LBCL, an heterogeneous group of malignancies with varying clinical behaviors and different pathological and molecular features, providing a comparison between the two classifications. Besides the well-recognized diagnostic role of clinical, morphological and immunohistochemical data, both classifications recognize the ever-growing impact of molecular data in the diagnostic work-up of some entities. The main aim is to offer a guide for clinicians and pathologists on how the new classifications can be applied to LBCL diagnosis in routine practice. In the first part of the paper, we review the following categories: LBLs transformed from indolent B-cell lymphomas, diffuse large B-cell lymphoma, not otherwise specified (DLBCL, NOS), double-hit/triple-hit lymphomas (DH/TH), high-grade large B-cell lymphoma, not otherwise specified (HGBCL, NOS), LBCL with *IRF4* rearrangement, Burkitt lymphoma (BL) and HGBCL/LBCL with *11q* aberration, focusing on the differences between the two classifications. In the second part of the paper, we provide a practical diagnostic algorithm when facing LBCLs in routine daily practice.

## 1. Introduction

The World Health Organization (WHO) classification of hematolymphoid tumors, edited first in 2001 and, subsequently, in 2008 and 2016, has been internationally used for diagnosis and has been recently replaced by both the fifth edition of the WHO classification (referred to as WHO-HAEM5) and the International Consensus Classification (ICC) [1,2]. Although both WHO-HAEM5 and ICC are built on the same idea, that is the definition of a disease entity based on clinical, pathological and, if available, molecular features, differences between the two classifications exist.

For instance, unlike ICC, provisional entities are not accepted in WHO-HAEM5, whereas some entities considered provisional in the previous WHO classification (referred to as WHO-HAEM4) [3] are now upgraded to definite categories in both ICC and WHO-HAEM5 (Table 1).

Diagnostic criteria and suggested ancillary analyses have been defined in both classifications and, in particular, in WHO-HAEM5, where a practical approach with “essential” and “desirable” diagnostic criteria for each entity is used. For the first time, in WHO-HAEM5, a chapter on transformations of indolent B-cell lymphomas is included.

The purpose of this paper is to compare the two recent classifications in terms of disease definition and diagnostic criteria, focusing on large B-cell (LBCL) non-Hodgkin lymphomas, which are a very heterogeneous family of tumors with varying clinical behaviors and different pathological and molecular features.

In our paper, we discuss the entities included in the large family of LBCL, focusing on the following diseases: LBLs transformed from indolent B-cell lymphomas, diffuse large B-cell lymphoma, not otherwise specified (DLBCL, NOS), double-hit/triple-hit lymphomas (DH/TH), high-grade large B-cell lymphoma, not otherwise specified (HGBCL, NOS), LBCL with *IRF4* rearrangement, Burkitt lymphoma (BL) and HGBCL/LBCL with 11q aberration, pointing out, in particular, the differences between ICC and WHO-HAEM5. In the last part of the paper, we describe a practical diagnostic approach when facing aggressive peripheral B-cell lymphoma diagnosis in pathologist’s daily practice. The diagnostic algorithm applies to any nodal and extranodal aggressive peripheral B-cell lymphoma not fulfilling the diagnostic criteria of any specific LBCL entity. Therefore, specific entities such as EBV-positive DLBCL, primary mediastinal large B-cell lymphoma (PMLBCL), T-cell/histiocyte-rich large B-cell lymphoma (TCHRLBCL), ALK-positive LBCL, plasmablastic lymphoma (PBL), primary effusion lymphoma (PEL), HHV8-positive LBCL, lymphomatoid granulomatosis (LYG), DLBCL associated with chronic inflammation, fluid-overload-associated LBCL, fibrin-associated LBCL, mediastinal grey zone lymphoma (MGZL), intravascular LBCL, primary LBCLs of immune-privileged sites and primary cutaneous DLBCL and leg-type 2 are not discussed as the discussion of these entities goes beyond the aim of the present paper.

## 2. Transformation of Indolent B-Cell Lymphomas

This section has been introduced for the first time in the recent WHO-HAEM5, whereas this issue has not been addressed in previous WHO classifications nor in ICC. Transformation is defined as the occurrence of an aggressive lymphoma in a patient with a previous or synchronous clonally related, indolent B-cell lymphoma. Transformation to an aggressive lymphoma is part of the natural course of various indolent lymphomas such as chronic lymphocytic leukemia/small lymphocytic lymphoma (CLL/SLL), marginal zone lymphoma (MZL), follicular lymphoma (FL) and lymphoplasmacytic lymphoma (LPL) despite occurring with a different frequency. Transformation should be suspected by the appearance or worsening of systemic symptoms, increasing LDH levels and rapidly enlarging lymph nodes with high PET uptake. The clinical suspicion of transformation should always be confirmed by histology. LBLs transformed from indolent B-cell lymphomas are indistinguishable from the de novo forms of LBCL, which will be discussed in the following paragraphs. Generally, the aggressive lymphoma retains the same immunophenotype of the indolent form, such as the expression of CD5 and CD23 in cases transformed from CLL. An aggressive lymphoma, clonally unrelated to the indolent B-cell lymphoma, is not considered a transformed lymphoma but a de novo secondary malignancy occurring in a patient with a history of indolent B-cell lymphoma. Cases of clonally related aggressive lymphoma carry a more aggressive course compared to de novo, clonally unrelated diseases. Therefore, the demonstration of the clonal relationship between the aggressive lymphoma and the indolent form is essential [1,4,5].

## 3. Diffuse Large B-Cell Lymphoma, Not Otherwise Specified (DLBCL, NOS)

### 3.1. General and Histological Features

DLBCL, NOS is an aggressive B-cell lymphoma, which does not meet the diagnostic criteria for any of the specific LBCL entities [1,2]. DLBCL, NOS involves lymph nodes, and, in 30–40% of cases, the disease is extranodal at presentation.

Histologically, it consists of a diffuse proliferation of medium to large cells more often with a centroblastic or immunoblastic morphology, although other morphologic variants of the disease, such as the anaplastic variant with more pleomorphic cells, are well known. Data on the correlation between morphological variants and prognosis are rather scarce.

Tumor cells express pan B-cell markers (CD20, CD79 alpha, PAX5 and CD19) and CD45 and, generally, surface immunoglobulin. Other markers used to characterize DLBCL, NOS are CD10, BCL6, BCL2 and IRF4/MUM1. The expression of CD5 may be found in 5–10% of DLBCL, NOS; the reported adverse prognostic impact of CD5+ DLBCL, NOS is likely related to their activated B-cell (ABC) type [1]. The ICC de-emphasize the relevance of CD5+ DLBCL, NOS because it does not represent a distinct biological group [2]. CD5+ DLBCL can be differentiated from pleomorphic and blastoid mantle cell lymphoma (MCL) by the absence of cyclin D1 and SOX11 [1,3]. Cyclin D1 may be expressed in 1–2% of DLBCL, NOS; however, these cases lack SOX11 and *CCND1* translocation [1,3].

### 3.2. Cell of Origin (COO) Classification Retained by Both ICC and WHO-HAEM5

An important aspect in DLBCL, NOS is the cell of origin (COO) classification.

Both ICC and WHO-HAEM5 recommend retaining the COO classification due to its potential prognostic impact [1,2].

The gene expression profiling (GEP) identified three subgroups in DLBCL, NOS: the germinal center B-cell (GCB) subtype (40–50%), the activated B-cell (ABC) subtype (50–60%) and the unclassified subtype (10–15%) [6,7]. GCB-DLBCL is composed of large cells, often resembling centroblasts, recapitulating the differentiation mechanisms occurring in normal germinal centers (GCs) with an expression of GC immunohistochemical markers (CD10 and BCL6), hypermutated immunoglobulin, and ongoing somatic hypermutation. ABC-DLBCL consists of large cells, often with immunoblastic features, and has a gene profile of post-GCBs with a lack of GC markers, an expression of MUM1/IRF4 and a constitutive activation of BCR signaling and nuclear factor kappa B (NF-kB) pathways.

The ABC subtype has a worse prognosis in response to standard therapies compared to the GCB subtype [6,7,8,9].

GEP is recognized as the gold standard for subtyping DLBCL, NOS according to the COO classification. However, GEP has not been routinely introduced, and several immunohistochemical algorithms to reproduce the molecular COO classification have been developed. Immunohistochemistry-based algorithms are used by pathologists [1,10,11,12] in daily practice because they are simple, rapid and low cost and, unlike GEP, do not need fresh or frozen (FF) samples. However, immunohistochemical algorithms are not completely reliable both for the variable concordance with GEP and for the subjectivity among pathologists in immunohistochemical result interpretation. The Hans and Tally algorithm, based on the immunohistochemical expression of CD10, BCL6 and MUM1/IRF4, is the most-applied method to define the COO [1,10], but concordance between GEP and Hans algorithm is moderate (72–86%) [13,14]. For instance, cases positive for CD10 and MUM1 are by default classified as GCB type by the Hans algorithm, but, by GEP, 30–50% of these cases belong to the ABC type [15]. Other immunohistochemistry-based algorithms, such as the Tally and Choi assays, have a higher concordance with GEP compared to the Hans algorithm [11,16]. However, these methods are not widely used because of some technical issues with LMO2, FOXP1 and GCET1 [11,16].

Customized GEP applied on the NanoString platform to formalin-fixed, paraffin-embedded (FFPE) samples represents a more reliable technique for predicting prognosis compared to immunohistochemistry-based algorithms [13,14].

Nevertheless, it is clear that, even with GEP, the COO classification does not fully capture the biological complexity of DLBCL, NOS, and there is the need to move to a molecularly-based classification of the disease. High-throughput techniques, such as next-generation sequencing (NGS) analyzing a large number of DLBCL, NOS, revealed a heterogeneous molecular landscape, and several groups have proposed different molecular subtypes of DLBCL, NOS with prognostic implications [17,18,19,20]. As already mentioned, ICC and WHO-HAEM5 recommend retaining the COO classification with the expectation that a unified molecular classification that is routinely applicable will be feasible in the near future.

### 3.3. MYC and BCL2 Immunohistochemical Expression and Double Protein Expresser (DPE) DLBCL, NOS

The immunohistochemical expression of MYC protein in DLBCL, NOS is highly variable. In the majority of the studies, MYC is considered positive if = or >40% of tumor cell nuclei are positive [21,22,23,24]. BCL2 is expressed in most DLBCL, NOS, and the cutoff level for BCL2 positivity is set to = or >50% of the tumor cells being BCL2 positive [25,26]. Some DLBCLs, NOS express both MYC and BCL2 by immunohistochemistry, without the presence of the rearrangement of these genes. Double protein expresser (DPE) cases, without genetic double-hit (DH), can be found in ABC-DLBCL, NOS, whereas the majority of molecular DH cases are of GCB type. Concurrent BCL2 and MYC protein expression has been reported to be associated with an adverse outcome [25,26]. However, clinical trials demonstrated that DPE cases of GCB type had indeed a poor prognosis, but this was not seen in DPE cases of ABC type [27]. Other studies demonstrated that the worse outcome associated with DPE was likely due to other elements such as TP53 alterations [28]. ICC de-emphasizes the prognostic significance of DPE taken by itself [29].

The term DPE should not be used as a synonym of true DH lymphomas.

### 3.4. FISH Analysis in DLBCL

Fluorescence in situ hybridization (FISH) analysis for *MYC*, *BCL2* and *BCL6* is strongly recommended in all DLBCL cases by both WHO-HAEM5 and ICC in order to identify a more aggressive subset of LBCLs.

Based on morphology and phenotype, these cases would be regarded as DLBCLs, NOS, but the identification of genetic rearrangements of *MYC*, *BCL2* and *BCL6* by FISH analysis allows one to separate this category of aggressive lymphomas with a poorer clinical outcome with standard R-CHOP and clinical consideration for intensified chemotherapeutic strategies.

In the previous 2017 WHO classification (WHO-HAEM4), these lymphomas, known as DH or triple-hit (TH) lymphomas, were placed in a provisional category of high-grade B-cell lymphomas with *MYC* and *BCL2* and/or *BCL6* rearrangements (HGBCL-DH/TH) [3].

Currently, the WHO-HAEM5 classification recognizes as a definite entity DLBCL/HGBCL with *MYC* and *BCL2* rearrangement (with or without *BCL6* rearrangement) [1].

According to WHO-HAEM5, cases carrying either an isolated *MYC* rearrangement or *MYC* and *BCL2* abnormalities other than typical translocations or dual *MYC* and *BCL6* rearrangements should be placed in the category either of DLBCL, NOS or HGBCL depending on their morphology.

The ICC recognizes two groups of DH-HGBCL: HGBCL with *MYC* and *BCL2* rearrangement (with or without *BCL6* rearrangement) (HGBCL-DH-BCL2) and the provisional entity named HGBCL with *MYC* and *BCL6* rearrangement (HGBCL-DH-BCL6) [2].

Aggressive B-cell lymphomas with the genetic rearrangements of *MYC, BCL2* and *BCL6* may show either a blastoid morphology or an intermediate morphology between Burkitt lymphoma (BL) and DLBCL or, in half of cases, may be morphologically indistinguishable from DLBCL, NOS. Therefore, FISH analysis is critical in all cases of LBCL.

## 4. DLBCL/HGBCL with *MYC* and *BCL2* Rearrangements (DLBCL/HGBCL-*MYC/BCL2*) in WHO-HAEM5 Named HGBCL with *MYC* and *BCL2* Rearrangements (HGBCL-DH-BCL2) in ICC

DLBCL/HGBCL-MYC/BCL2 is the only category of DH/TH currently recognized in WHO-HAEM5. It represents 80–90% of DH/TH lymphoma cases, and it characterized by structural chromosomal aberrations with breakpoints at both *MYC* and *BCL2* loci. Patients, mainly adults in the sixth or seventh decades of live, are often diagnosed at advanced stages. The aggressive behavior of this lymphoma may be due to the aberrant expression of BCL2, causing increased apoptotic resistance, and *MYC* activation, causing proliferation. *BCL2* (18q21) is translocated to IGH (14q32), whereas *MYC* (8q24) translocation may be with different partners, either immunoglobulin (IG) or non-IG [18,30,31]. *MYC* is translocated to IG loci in about 55% of cases, mainly IGH or alternatively IGL (8;22)(q24;q21) and, more rarely, IGK (2;8)(p11;q24). Non-IG partners of *MYC* translocation may be for instance BCL6 and PAX5 [30,32].

The clinical outcome of this category appears to be influenced by the partners of *MYC* translocation. Some studies have demonstrated that DLBCL/HGBCL-MYC/BCL2 with *IG::MYC* has an inferior survival compared to cases with non-*IG::MYC* [31,33]. However, others showed discrepant results as there was no difference in survival between cases with IG versus non-IG rearrangements [34].

This entity is sometimes preceded by a history of FL.

Histologically, this lymphoma may have a DLBCL morphology, hence is indistinguishable from DLBCL, NOS if FISH is not performed.

Some cases show a blastoid morphology resembling lymphoblasts, and other cases have a morphology intermediate between BL and DLBCL, with medium to large cells often with starry-sky macrophages. The majority (>90%) of these tumors have a GCB-like phenotype by the Hans algorithm or GEP analysis; CD10, BCL6 and BCL2 are positive in most cases, while MUM1 is generally negative. The immunohistochemical expression of MYC is found in the majority of cases using the cutoff of =/>40%. Approximately 71–81% of cases result in DPE of BCL2 and MYC [35]. The proliferative index is commonly high; however, a low index of proliferation does not rule out this entity [1,36].

The rather uniform phenotype of DLBCL/HGBCL-MYC/BCL2 supports their origin from an FL-like clone. The mutations often observed in DLBCL/HGBCL-MYC/BCL2 such as mutations in *BCL2*, *CREBBP*, *EZH2* and *TNFRSF14* overlap with the mutational profile of FL and DLBCL-GCB type [37]. Mutations in *ID3* and *CCND3*, common in BL, are also found in DLBCL/HGBCL-MYC/BCL2 [37]. *TP53* mutations are often found in this category in contrast to cases with concomitant *MYC* and *BCL6* rearrangements.

Rare cases with blastoid or intermediate morphology may show variable TdT expression ranging from rare positive cells to diffuse positivity. TdT expression is found in 11–14% of DH/TH cases [38,39], and the identification of TdT by itself is not currently considered sufficient to classify these cases as B-lymphoblastic leukemia/lymphoma (B-ALL/LBL).

Unlike WHO-HAEM4, both WHO-HAEM5 and ICC recommend classifying these cases as DLBCL or HGBCL with TdT expression. Of note, these cases show a mature B-cell phenotype, often with surface IG light chain expression, and never express CD34 [37,38]. If CD34 is positive, this should prompt consideration for B-ALL/LBL. They need to be differentiated from B-ALL with *MYC* rearrangement, which is a very rare entity occurring in children and young adults [38,39].

The correct identification of DLBCL/HGBCL-*MYC*/*BCL2* is essential for the prognostic and therapeutic impact. This entity usually follows an aggressive course, and treatments with standard R-CHOP regimen are insufficient with a 5-year survival rate of 40–50%; therefore, dose-intensified therapies are needed, especially in younger patients [40].

## 5. HGBCL with *MYC* and *BCL6* Rearrangement (HGBCL-DH-BCL6) Recognized in ICC Classification as Provisional Entity

WHO-HAEM5 has redefined the category of DH/TH, excluding cases with concomitant *MYC* and *BCL6* rearrangements as these cases are genetically heterogeneous. According to WHO-HAEM5, cases carrying dual *MYC* and *BCL6* rearrangements fall within DLBCL, NOS or HGBCL depending on their morphological features [1].

HGBCL-DH-BCL6 is currently considered a provisional entity in 2022 ICC, and it accounts for the minority (10–20%) of DH cases [2]. Patients are usually of 60–70 years, and extranodal sites are more often involved. Data on prognosis are rather controversial because some clinical trials confirmed a poor outcome, whereas others demonstrated an outcome similar to DLBCL, NOS [31,33,41,42]. Morphologically, cases may often resemble DLBCL and less frequently HGBCL [31,42]. Unlike the group carrying *MYC/BCL2* rearrangements, HGBCL-DH-BCL6 shows more frequently a non-GCB/ABC phenotype, being often MUM1-positive and CD10-negative. MYC is expressed in 90% of cases and BCL2 in 20–80% [31,42]. Interestingly, in this category, there is a subset of cases, approximately 30%, in which the *BCL6* gene is the *MYC* partner gene, and this group is termed pseudo-hit lymphoma [43,44]. Additional studies are essential to understand if the pseudo-DH tumors need to be separated from HGBCL-DH-BCL6. The mutational landscape of HGBCL-DH-BCL6 is much more heterogeneous than that of LBCLs carrying *MYC* and *BCL2* rearrangements. HGBCL-DH-BCL6 carry less frequently mutations recurrent in HGBCL-DH-BCL2 such as *CREBBP*, *KMT2D*, *EZH2*, *CCND3* and *ID3*, whereas they show mutations in genes such as *CD79B* and *PIM1,* which are associated with non-GCB DLBCL [1,37,45]. Of note, Kunstner et al. provided evidence of molecular divergences between HGBCL-DH-BCL2 and HGBCL-DH-BCL6, showing an enrichment of *SBS6* mutation in BCL6-rearranged cases; on the other hand, an impairment of *TP53* and *MYC* pathway, often observed in BCL2-rearranged cases, was lacking in BCL6-rearranged ones [45,46].

## 6. Burkitt Lymphoma (BL)

### 6.1. Clinical, Pathological and Molecular Features

The basic features of the disease remain unchanged in both WHO-HAEM5 and ICC. BL is a mature aggressive B-cell lymphoma in which classically three clinical subtypes have been described, namely, endemic (eBL), sporadic (sBL) and immunodeficiency-related BL (iBL) [44,46,47]. BL involves predominantly extra-nodal sites with differences among the three subtypes.

eBL occurs mainly in children, from equatorial areas in sub-Saharan Africa and South America, with a male predominance, presenting with rapidly growing masses involving mainly the jaw and facial bones in younger children and the ileocecal region in older children.

sBL is observed worldwide, mainly in children and young adults, although three peaks of age at 10, 40 and 70 years are observed. In sBL, the disease again prefers extranodal sites, more frequently the abdomen. Lymph node presentation is rare but reported more often in adults.

iBL is seen more frequently in the setting of HIV but may occur also in transplant recipients and individuals with congenital immunodeficiency. iBL shows nodal and bone marrow (BM) involvement more often than the other subtypes.

BL is the first example of a virus-associated human neoplasm, being strictly associated with Epstein Barr virus (EBV), and shows the activation of the *MYC* oncogene [46]. The strength of EBV association with BL is different among the three subtypes of BL with a higher association found in eBL (95%) compared with sBL (20–30%) and iBL (25–40%) [47,48,49,50]. In eBL, EBV plays an important etiologic role, in synergy with a polymicrobial environment, in particular with *Plasmodium falciparum* [51,52].

Regardless of the clinical subtype, the histopathological features of BL are similar, and the disease typically consists of a diffuse proliferation of uniform, medium-sized cells with round nuclei and multiple small nucleoli. The cytoplasm is scant to moderate and, on Giemsa staining, is deeply basophilic with lipid vacuoles. Numerous mitoses are evident, and often a starry sky pattern reflecting the high proliferative index is seen.

Some cases, especially in HIV-positive patients, show a plasmacytoid differentiation with EBV positivity, and, interestingly, rare cases show numerous epithelioid histiocytes and granulomas, which may lead one to misinterpret the disease as an inflammatory/infectious process, particularly in small biopsies. These cases are often EBV-positive and follow a more favorable course [53].

BL-cells have a B-cell phenotype with strong positivity for CD20, CD79alpha, PAX5, CD19 and IgM expression with light chain restriction. BL is typically positive for GC markers (CD10 and BCL6) and is usually negative or rarely weakly positive for BCL2. The proliferative index is very high (ki67 > 95%), and there is strong MYC protein overexpression. EBV in situ hybridization (EBER) is positive in the majority of eBL and in about 30% of sBL and iBL [47,48,49,50,54].

The molecular hallmark of the disease is the *IG::MYC* translocation, juxtaposing *MYC* to an IG locus, commonly IGH (80%) by the t(8;14)(q24;q32) or, more infrequently, IGK (15%) and IGL (5%) by the t(2;8)(p12;q24) and the t(8;22)(q24;q11) [1].

Of note, recent studies suggested that EBV-positive BL and EBV-negative BL represent two distinct groups independently of the epidemiological and geographic context [1,55,56,57,58]. Hence, WHO-HAEM5 emphasizes the distinction of BL in two types: EBV-positive BL and EBV-negative BL, suggesting a dual mechanism of pathogenesis: mutational versus virus-driven [1,52]. EBV-positive BL carries higher levels of somatic hypermutations and fewer driver mutations, particularly in the apoptosis pathway than EBV-negative BL, supporting the role of EBV in lymphomagenesis.

ICC recommends that cases previously reported as TdT-positive BL should be separated from BL and diagnosed as B-ALL/LBL with *MYC* rearrangement rather than BL as these neoplasms show immunophenotypic and molecular features of precursors B-cells [2,59].

Currently, intensive multi-agent chemotherapies have significantly improved the overall survival of BL patients to almost 90% of cases [60,61].

### 6.2. BL: Diagnostic Algorithm and Differential Diagnoses

The diagnosis of BL requires the following:

- Typical histology (a diffuse growth pattern of monomorphic, medium-sized cells with basophilic cytoplasm and multiple small nucleoli)
- Typical immunophenotype (positivity for B-cell markers, CD10, BCL6 and MYC; negativity (or rarely weakly positivity) for BCL2; negativity for TdT and cyclin D1; high ki67 >95%)
- *IG::MYC* translocations (an absence of *BCL2* or *BCL6* translocations).
- The differential diagnoses of BL include the following:
- B-ALL/LBL has a characteristic morphology (cells with scant cytoplasm and absent or barely visible nucleoli) and immunophenotype (an expression of markers of immaturity: TdT and CD34).
- Blastoid mantle cell lymphoma, which has classical immunophenotype (cyclin D1 and SOX11 expression and not always CD5 positivity) and molecular features (a presence of translocation t(11;14)(q13;q32).
- HGBCL, NOS may have histological features and *MYC* rearrangement similar to BL. Unlike BL, HGBCL usually is CD10-negative and BCL2 diffusely positive.
- DH/TH lymphomas (the presence of *MYC* rearrangement requires the exclusion of *BCL6* and *BCL2* rearrangements, especially in adult cases).
- HGBCL with 11q aberration. The cells may be BL-like, although usually more pleomorphic than BL; the immunophenotype is similar to BL (positivity for CD10 and BCL6 and negativity for BCL2); however, LMO2 is positive in 50% of HGBCL with 11q aberration and usually negative in BL). Moreover, *MYC* translocation is absent, and chromosome 11q gain/loss is identified.

## 7. HGBCL with 11q Aberration in WHO-HAEM5 (LBCL with 11q Aberration in ICC)

### 7.1. Clinical, Pathological and Molecular Features

WHO-HAEM4 recognized a provisional entity defined Burkitt-like lymphoma with 11q aberration, which showed some overlapping features with BL, but carried 11q aberration, in the absence of *MYC* rearrangement [3]. Currently, in WHO-HAEM5, the disease has been upgraded to definite entity under the name HGBCL with 11q aberration, whereas ICC still recognizes this entity as provisional under the name LBCL with 11q aberration [1,2]. The disease presents more frequently in children and young adults with localized nodal or extranodal involvement more often in the head and neck region or in the gastrointestinal tract. It is reported in the setting of HIV infection and in post-transplant patients or in individuals with primary immunodeficiency syndromes [62]. Morphologically the cohesive neoplastic proliferation consists of cells either resembling BL or with an intermediate morphology between BL and DLBCL or with a blastoid morphology. Compared to BL, the cells are more pleomorphic and variable in size and shape. A starry sky pattern is often present; in particular, the identification of coarse apoptotic debris is a rather characteristic morphological finding that may lead one to suspect the diagnosis [63,64]. The phenotype is similar to BL with the expression of CD10 and BCL6 and negativity for BCL2. LMO2 may be a useful marker, being usually negative in BL and positive in about 50% of HGBCL with 11q aberration [62]. MYC protein is variably expressed, although often weakly positive, whereas *MYC* rearrangement is by definition absent. EBV is not associated with the disease. The genetic hallmark of the disease is a complex aberration involving the long arm of chromosome 11 with a minimal region of gain in 11q23.3 and a minimal region of loss at 11q24.1. Some cases show a telomeric loss or telomeric loss of heterozygosity (LOH). The mutational profile of the disease is closer to DLBCL rather than to BL as HGBCL with 11q aberration generally lacks mutations found in BL, such as *ID3*, *TCF3* and *CCND3*. ICC has preferred the term LBCL with 11q aberration instead of HGBCL with 11q aberration due to the mutational profile similar to DLBCL of GCB type and the good outcome of the disease compared to the category of HGBCL [64,65,66].

The prognosis is good in young patients treated with therapeutic schemes applied in BL, whereas it is more variable in adults undergoing therapies for DLBCL [65,67].

### 7.2. HGBCL with 11q Aberrations: Diagnosis and Practical Issues

In order to correctly identify the disease, FISH analysis with an 11q probe should be performed in all cases with typical morphology (BL-like, intermediate or blastoid) and immunophenotype (GCB-phenotype with BCL2 negativity), lacking *MYC* rearrangement, presenting in young patients in particular.

FISH analysis is the test more commonly used, although it cannot detect all genomic alterations. Currently, the presence of an 11q23.3 gain alone, in the absence of a concomitant 11q24 loss, is not considered sufficient for defining HGBCL with 11 q aberrations. In such cases, more sophisticated techniques, such as copy number analysis by array and next-generation sequencing (NGS) analyses, are needed to identify the genomic alterations, such as telomeric LOH [68].

It is well known that cases of classic *MYC*-rearranged BL may acquire 11q aberrations as a secondary event; these cases are by definition excluded from the category of HGBCL with 11q aberrations due to the presence of *MYC* rearrangement. However, rare cases of HGBCL with 11q aberrations may acquire *MYC* rearrangement as a secondary alteration during the disease course [61,69,70]. How to deal with such cases is an open question for clinicians and pathologists.

## 8. Large B-Cell Lymphoma (LBCL) with *IRF4* Rearrangement

LBCL with *IRF4* rearrangement was first introduced as a provisional entity under FL in WHO-HAEM4, and it is still retained there in ICC [2,3]. In WHO-HAEM5, it has been moved to the group of aggressive B-cell lymphoma [1]. It is a rare de novo LBCL, presenting usually in children and young adults with a localized disease involving commonly the Waldeyer ring and cervical nodes and, more rarely, other sites. The outcome is excellent. Histologically, it shows a follicular, diffuse or combined follicular and diffuse pattern of growth. It is composed by large cells with a centroblastic appearance and, more rarely, by smaller cells with a blastoid morphology. Neoplastic cells express a mature B-cell phenotype with characteristic strong positivity for both BCL6 and MUM1, whereas CD10 and BCL2 are expressed in half of the cases. The strong MUM1 expression is usually due to a *IG::IRF4* translocation, most often with the IGH and rarely with IGK and IGL [71,72]. *BCL2* and/or *MYC* rearrangements are absent and aggressive B-cell lymphomas with *IRF4* rearrangement, harboring also *MYC* and/or *BCL2* rearrangements, are excluded from this entity.

LBCL with IRF4 rearrangement needs to be differentiated from pediatric-type FL (PTFL), which, analogously to LBCL with IRF4 rearrangement, presents in the same group of age as a localized disease often in the head and neck area [73]. However, in PTFL, extranodal presentation is excluded; the pattern of growth is purely follicular, and diffuse areas of large cells meeting criteria for LBCL are excluded by definition. Neoplastic cells show a GC phenotype with the absence of a strong, uniform MUM1 expression, and *IRF4*, as well as *MYC*, *BCL2* and *BCL6*, rearrangements are absent.

## 9. High-Grade B-Cell Lymphoma, Not Otherwise Specified (HGBCL, NOS)

### 9.1. Clinical, Pathological and Molecular Features

HGBCL, NOS is a rare, heterogeneous entity characterized by cells with either a blastoid morphology or with features intermediate between BL and DLBCL, NOS, not fulfilling the diagnostic criteria for other well-defined entities [1,2,36,74,75].

Clinically, it occurs mainly in elderly individuals presenting with a widespread involvement of nodal and extranodal sites. Histologically, it consists of a diffuse proliferation of cells with intermediate morphology and usually more pleomorphism than in BL; in other cases, the cells show a blastoid appearance, being small to medium in size with scarce cytoplasm, inconspicuous nucleolus and dispersed chromatin. A starry-sky pattern may be found. It often shows a GCB phenotype with the expression of CD10, BCL6 and BCL2; a minority of cases is non-GCB. MYC protein is variably expressed. Approximately 40% of cases are DPE lymphomas, being positive for both MYC and BCL2 proteins [76,77].

By definition, the diagnosis of HGBCL, NOS is excluded when *MYC* rearrangement is found in association with *BCL2* rearrangement and when 11q aberrations are identified.

According to WHO-HAEM5, cases with HGBCL morphology harboring *MYC* and *BCL6* rearrangement are included in the category of HGBCL, NOS.

The diagnosis of HGBCL, NOS is actually a diagnosis of exclusion, and the interobserver variability is well recognized in diagnosing this entity [78]. Therefore, this diagnosis should be made only when the case does not fulfill the diagnostic criteria of BL, DLBCL and other well-defined entities.

Limited data on the molecular features of this entity are reported. Isolated *MYC* rearrangements are described in 8–58% of cases and *MYC* amplification in 32% [79]. Other molecular abnormalities identified in HGBCL, NOS are the following: *MYC* amplification concurrent with *BCL2* translocation; the amplification of both *MYC* and *BCL2*; translocations of *BCL2* and *BCL6* [80]. A recent study by Li et al. showed that a subset of HGBCL, NOS is genetically much closer to DLBCL/HGBCL-*MYC/BCL2* than to DLBCL, NOS [76].

### 9.2. HGBCL, NOS and Differential Diagnoses

As mentioned above, HGBCL, NOS is diagnosed after excluding other well-defined entities.

The differential diagnoses should include the following diseases:

- B-ALL/LBL express markers of immaturity such as TdT and CD34.
- The blastoid variant of MCL express cyclin D1 and SOX11. Of note, the blastoid variant of MCL may show some variations of the immunophenotype such as a loss of CD5 (25–28% of cases) and an aberrant expression of CD10 and BCL6, making difficult the correct diagnosis [77,81,82].
- BL should be excluded on the basis of morphological, immunophenotypic and molecular features. In particular, a variation in size and shape of the nuclei is still accepted in BL if combined with the classic BL immunophenotype (CD10+, BCL6+ and BCL2-) and isolated *MYC* translocation. On the other hand, cases with strong BCL2 expression have been demonstrated to have frequent cytogenetic abnormalities and are better classified as HGBCL, NOS [70].
- HGBCL-*MYC/BCL2* and HGBCL with 11 q aberrations should always be excluded by FISH analysis.

## 10. Practical Diagnostic Algorithm in Large B-Cell Lymphomas

The diagnostic algorithm should be applied to any aggressive peripheral B-cell lymphoma not fulfilling the diagnostic criteria of well-defined entities recognized in both WHO-HAEM5 and ICC (see Table 1). For instance, cases of EBV-positive DLBCL, similarly to other well-defined entities, are excluded from the diagnostic workflow.

The initial diagnostic approach of aggressive peripheral B-cell lymphomas should include the evaluation of the morphological and immunophenotypic characteristics including, as previously discussed, the COO and the percentage of MYC and BCL2-positive neoplastic cells.

Prior to the identification of DH/TH lymphomas, FISH for *MYC* was performed at the pathologist’s discretion only in cases with high-grade morphology or a high ki67 proliferation index. However, it is now well proven that high-grade morphology is inadequate for screening for *MYC* rearrangement as about 10% of morphologically typical DLBCLs carry *MYC* rearrangement [83].

As such, currently both WHO-HAEM5 and ICC strongly recommend screening all LBCLs with FISH analysis for *MYC*, *BCL2* and *BCL6* in order to identify more aggressive subsets of LBCLs requiring dose-intense treatment protocols, which are usually adopted in fit and young patients.

Treatment choice is also driven by patient fitness, including age and comorbidities. Therefore, in order to save resources, before proceeding with FISH analysis, it is recommended to discuss the cases with the referring hematologist to verify the patient clinical condition and potential eligibility to intensified chemotherapy [73,84].

- Having said that, in general, FISH analysis would be advisable not only in LBCLs with a blastoid or intermediate morphology but even in cases of otherwise morphologically typical DLBCL.
- As mentioned in the previous paragraphs, in cases of B-cell lymphomas with blastoid morphology, first of all, the blastoid variant of MCL and B-ALL/LBL needs to be excluded with the appropriate immunohistochemistry (cyclin D1 and TdT, respectively). Of note, the use of FISH to detect *CCDN1* rearrangement can be helpful to distinguish the blastoid variant of MCL from the rare cases of cyclin D1+ LBCLs. In LBCLs with blastoid features, FISH analysis for *MYC*, *BCL2* and *BCL6* allows the classification of the disease as either HGBCL/DH-TH or HGBCL, NOS.
- In cases with morphological features intermediate between BL and DLBCL, FISH analysis for *MYC*, *BCL2* and *BCL6* rearrangements allows the correct differential diagnosis among BL, DH/TH lymphomas and HGBCL, NOS. In cases lacking *MYC* translocation, FISH analysis for 11q aberrations should be performed in order to correctly identify the category of HGBCL with 11q aberration.

## 11. FISH Probe Strategy to Apply in LBCLs

Rearrangements of *MYC*, *BCL2* and *BCL6* are commonly assessed using FISH breakapart (BA) probes. Since by definition, DH/TH lymphomas require the identification of *MYC* rearrangement, this should be explored first, followed by *BCL2* and *BCL6* gene study.

In LBCLs, the initial screening approach typically begins with a sensitive *MYC* FISH BA probe. Of note, commercially available *MYC* BA FISH probes may differ significantly in sensitivity, depending on the extent of the breakpoint region covered in the probe [85,86]. However, even the most sensitive *MYC* BA probes may not be able to detect some rearrangements due to cryptic insertions or other genomic rearrangements that are out of the coverage area of the probes. Therefore, the use of *MYC::IGH* dual-fusion (DF) FISH, in addition to *MYC* BA, may increase the sensitivity of detection, identifying from 4% to 12% of *MYC* rearrangements, which are missed by BA alone [83,86]. The sensitivity in identifying *MYC* rearrangements might be increased by adding *MYC::IGL* and *MYC::IGK* probes; however, data are not available because these probes are rarely used [86].

It is also well defined that in BL, the *MYC* partner is almost always an IG partner, whereas in non-BL LBCLs, the *MYC* partner genes include several non-IG partners in approximately 40% of cases; consequently, a part of *MYC* rearrangements are undetectable by FISH analysis, and, currently in research, other molecular methods are used [87]. As previously mentioned, the definition of the partner gene of *MYC* (either IG or non-IG) might be clinically relevant as cases with *MYC::IG* usually have an inferior survival compared to those with *MYC*-non IG translocations, but this issue is still under debate [31,33,34]. Currently, both WHO-HAEM5 and ICC do not require the identification of the *MYC* partner gene.

In addition, both classifications state that the identification of the copy number alteration (CNA) of *MYC*, *BCL2* and *BCL6* is not equivalent to gene rearrangements for classifying LBLCLs as DH/TH [1,2]. The significance of such extra copies remains to be clarified as some studies demonstrated that the CNA of *MYC* is associated with poor prognosis, while others found discrepant results [80,88,89,90].

## 12. Conclusions

The accurate diagnosis of clinically distinct forms of LBCL is essential for the choice of the adequate therapy. Currently, both WHO-HAEM5 and ICC state that the precise classifications of these entities is based on the combination of morphological, immunophenotypical and molecular features. Despite some differences between WHO-HAEM5 and ICC, which have been highlighted in the present paper, the application of the FISH strategy is highly recommended by both classifications in order to identify cases that may benefit from intensified chemotherapeutic regimens.

## Figures and Tables

**Table 1:** Large B-lymphomas in WHO-HAEM4, WHO-HAEM-5 and ICC.

WHO-HAEM4	WHO-HAEM5	ICC
Not included as entity	Transformations of indolent B-cell lymphomas	Not included as entity
DLBCL, NOS (COO recommended)	DLBCL, NOS (COO recommended)	DLBCL, NOS (COO recommended)
BL	BL (it is stressed the value of EBV positivity)	BL
HGBCL with *MYC* and *BCL2* and/or *BCL6* rearrangements	DLBCL/HGBCL with *MYC* and *BCL2* rearrangements	HGBCL with *MYC* and *BCL2* rearrangements
Not included as entity	Not included as entity (it is included in DLBCL/HGBCL, NOS depending on morphology)	HGBCL with *MYC* and *BCL6* rearrangements (provisional entity)
HGBCL, NOS	HGBCL, NOS	HGBCL, NOS
LBCL with *IRF4* rearrangement (provisional entity under FL)	LBCL with *IRF4* rearrangement (definite entity in the group of LBCL)	LBCL with *IRF4* rearrangement (definite entity under FL)
BL-like lymphoma with 11q aberrations (provisional entity)	HGBCL with 11q aberrations (definite entity)	LBCL with 11q aberrations (provisional entity)

BL: Burkitt lymphoma; COO: cell of origin; DLBCL, NOS: diffuse large B-cell lymphoma, not otherwise specified; HGBCL: high-grade B-cell lymphoma; ICC: International Consensus Classification; LBCL: large B-cell lymphoma; WHO-HAEM4: WHO classification, revised 4th edition; WHO-HAEM5: WHO classification, 5th edition.

## Data Availability

Individual patient data from the original studies included in the present review are not available, and data sharing at this level is not applicable for a review.
